# The Influence of Doctor-Patient and Midwife-Patient Relationship in Quality Care Perception of Italian Pregnant Women: An Exploratory Study

**DOI:** 10.1371/journal.pone.0124353

**Published:** 2015-04-23

**Authors:** Laura Andrissi, Felice Petraglia, Alessandro Giuliani, Filiberto Maria Severi, Stefano Angioni, Herbert Valensise, Silvia Vannuccini, Nunziata Comoretto, Vittoradolfo Tambone

**Affiliations:** 1 Institute of Philosophy of Scientific and Technological Activity, University Campus Bio-Medico, Rome, Italy; 2 Department of Molecular and Developmental Medicine, Obstetrics and Gynecology, University of Siena, Siena, Italy; 3 Environment and Health Dept. Istituto Superiore di Sanità, Rome, Italy; 4 Division of Gynaecology, Obstetrics and Pathophysiology of Human Reproduction, Department of Surgery, Maternal-Fetal Medicine and Imaging, University of Cagliari, Cagliari, Italy; 5 Department of Obstetrics and Gynecology, Tor Vergata University, Rome, Italy; National Institute of Health, ITALY

## Abstract

**Background:**

The study focuses on the perceived nature / technique opposition in pregnancy and delivery emerging from gynaecologist/ midwife/ pregnant woman relationships. We developed a cross-sectional survey to identify, by means of a multidimensional data-driven approach, the main latent concepts structuring the between items correlation correspondent to the different general opinions present in the data set. The obtained results can set the basis to improve patient satisfaction while decreasing healthcare costs.

**Methods:**

The sample is made of 90 pregnant women within 24-48 hours after natural or operative birth, from three maternity units in Italy. Women filled in a questionnaire about their relationship with gynaecologist and midwife during pregnancy and hospital stay for delivery.

**Results:**

Participation rate approached 100%. The emerging factorial structure gave a proof-of-concept of the hypothesis of ‘nature vs. technique’ as the main dimension shaping women opinions. The results highlighted the role of midwife as the ‘link’ between the natural and technical dimension of birth. The quality of welcome and the establishing of an empathic relation between mother and healthcare professional was shown to decrease further request of care in the post-partum period.

**Conclusions:**

The “fault plane” between nature and technique is a very critical zone for litigation. Women are particularly sensitive to the consideration and attention they receive at their admission in the hospital, as well as to the quality of human relationship with midwife. The perceived quality of welcome scaled with a decreased need of additional care and, more in general, with a more faithful attitude towards health professionals. We hypothesize that increasing the quality of welcome can exert an effect on both welfare costs and litigation. This opens the way (through an extension of this pilot study to wider populations) to relevant ameliorative actions on quality of care at practically null cost.

## Introduction

Childbirth as a medical act has a very recent origin. For most mankind history birth was considered as a natural and social event where mother was supported by intermediate figures at cross-roads between technical and familiar fields. The event of birth took place in a domestic and strictly feminine environment: female care providers guided woman through her labor.

From 20^th^ century, birth underwent a process of medicalization and pregnant woman begun to share the experience of pregnancy and delivery with care professional figures. However, maternity is still a personal and emotional experience and represents a major lifetime event; as such, women should have the opportunity to have a positive birth experience even in a medical environment [1,2]. Governments and professional recommendations have long determined that maternity services should take women individual needs into account and offer greater choices [3]. Besides, there is increasing attention to satisfaction with care and patient-centred healthcare, as key factor to improve outcome and decrease litigation [4].

The dissatisfaction expressed by patients became an object of increasing attention by health care organizations, not only because such dissatisfaction often translates into formal complaints and even lawsuits, but also because the perception of the patient is an important index for evaluating the quality of service provided. The analysis of the factors determining patient satisfaction, therefore, represents a relevant component of clinical governance in order to meet an important ethical requirement and to limit the risk of medico-legal litigation against the health care facility.

Several studies show that most complaints generate from interpersonal relationship of care [5–7]. The dissatisfaction depends both on the relational style of single professionals and on how the organizational system promotes or prevents the realization of an appropriate relationship between doctor and patient. Other studies [8] indicate that the majority of complaints involve problems of doctor-patient communication. Moreover, even when the complaint is formally presented in terms of dissatisfaction related to technical aspects of medical care, a deeper analysis reveals that the real motivation stems from the relational sphere [9]. Studies specific to obstetric environments [6] demonstrate quality of the relationship, the attitudes of practitioners and the environment of care are the most relevant factors influencing the perception of the quality of assistance. Other studies [10, 11] show that conciliatory mediation *per se* does not exert a positive effect on the prevention of episodes of complaints that, in addition, require a suitable support by the health care facility in order not to fall into defensive attitudes towards patients as a result of complaints received [12]. Bowling [13] points to the need of a careful analysis of the reasons that lead patients and their families to make a complaint and of the practical actions that can be effective in the prevention of situations that give rise to complaints.

Some studies, in turn, suggest that current methods of assessing/improving patient satisfaction are effective [14], but they are not the most productive.

The aim of the present study is to evaluate if the opposition nature / technique linked to the increasing needs of birth medicalization is only a fascinating intellectual construct or, on the contrary, a useful operational principle to rationalize patient-doctors frictions. In order to explore such hypothesis a first pre-requisite is to give a proof-of-concept that such opposition emerges spontaneously from the priorities and wishes expressed by the mothers.

If this opposition emerges in a data-driven mode by the correlation structure of different items responses, the harmonization of these two factors could be the basis for improving the satisfaction of the mother while in the same time making savings in healthcare costs. Thus, beside the investigation of the tenability of such nature / technique ‘fault-plane’ hypothesis, the present study aims to validate the questionnaire as an adequate tool to evaluate following “cheap” interventions in the field of hospitals’ governance and as a tool to empower the educational strategies for health professionals.

## Materials and Methods

### 1. Questionnaire development

A panel of experts developed the initial set of items of the questionnaire, relying upon literature reviews and interviews of midwives and gynaecologists. After the removal of overlapping items, 120 were suitable for the questionnaire pool. An expert panel, consisting of 2 midwives, 4 physicians and 2 methodologists (one statistician and one epidemiologist), discussed these items both from the point of content validity and from the viewpoint of construct coherence. After a discussion round a final 98-item questionnaire was tested for compliance in a small sample of mothers and then consented and approved for the pilot study.

### 2. Study setting and participants

The pilot cross sectional validation study follow the STROBE [17] guidelines. It was conducted in three maternity units in Italy (Siena, University Hospital “Le Scotte”; Rome, Fatebenefratelli Hospital; Cagliari, University Hospital San Giovanni di Dio), between June 2013 and September 2013. Two days after childbirth, 90 women (30 for each hospital) who attended the maternity ward were invited by the interviews (medical doctors in gynaecologic training) to participate to the study and asked to fill in the questionnaire. The eligibility criteria for participants were: to be Italian, primi-gravid, with at term physiological pregnancy, spontaneous labour and delivery or elective caesarean section (scheduled caesarean section for fetal breech presentation or previous caesarean section). The inclusion criteria were restricted only to deliveries without complications, to rule out surgical, medical, obstetrics and psychological problems that could influence the perception of the patient relatively to the nature/technical balance in delivery. The interviewers informed the mothers, in a previous counselling session, that completion of the questionnaire was voluntary and confidential.

### 3. Data Analysis Strategy

The first step of the analysis was filtering out the variables (items) with no relevant variability among respondents. The threshold was set at a value of Coefficient of Variation (CV = SD/Mean) lower than 0.1. Given we focused on the correlation structure of the items, variables with no relevant variability across women do not carry any relevant information for the study.

As second filtering step, the remaining variables were analysed by Oblique Principal Components (OPC, VARCLUS procedure in SAS [15] subdividing the items into homogenous clusters according to the target of a grouping characterized by the maximal within-cluster correlation and the minimal between-cluster correlation [16, 18].

The ‘centrality’ of each variable as for its own cluster corresponds to its squared correlation with the cluster first principal component (see S1 Table). The variables with an R-square lower than 0.20 (see S1 Table) with their own cluster were eliminated as singular. The variables with an R-square higher than 0.70 with their own cluster were summed up so to generate a composite summary variable. This last procedure corresponds to the well-known Cronbach’s alfa commonality, in which the internally consistent items point to the same latent concept and thus are added together to build a synthetic index [19].

A group of 49 variables (answers to different items) survived the filtering steps and was sub-divided into three groups: 8 external variables (EXT), 28 evaluation (EVAL) variables, 13 opinion variables (OP) (see S1 File). The external variables refer to demographic (age, place of origin, marital status, hospital, school degree) and clinical (type of delivery, weight, height, BMI) information. These external variables do not enter into the subsequent step of generation of principal components but are analysed as for their correlation with the components arising from the EVAL and OP subsets.

Evaluation (EVAL) variables describe the experience patients had during their stay in hospital for delivery, while opinion (OP) variables express the ideal model of care of the same patients.

The OP and EVAL variables were separately analysed by means of Principal Components Analysis (PCA) and the relevant components extracted and interpreted on the base of their loadings (correlation coefficients between items and components) distribution. Then OP and EVAL components were correlated each other in order to check the effect that general ideas have on the health care evaluation. All the analyses were carried out by SAS system v.8.1 and the analysed data set is reported in S1 Dataset.

### 4. Ethical considerations

Only patients who gave a written informed consent were enrolled in the study. Given this was a non invasive epidemiological study there was no necessity to obtain a vote from a local research ethics committee [20] as the Declaration of Helsinki authorizes [21], in any case the University Campus Biomedico research ethic committee gave us the permission (Prot.43/2013 ComEt CBM) before the start of the study.

All the data were specificallycollected for this study. The medical doctors in gynecologic training who conducted the interviews with the women provided us with anonymized data. Stefano Angioni, Herbert Valensise, Silvia Vannuccini personally interacted with patients for data collection.

## Results and Discussion

### 1. Filtering out low variance and singular items

The initial questionnaire structure gave rise to 189 atomic variables (the increased number with respect to the initial 98 items comes from the fact multiple answer items were further subdivided into yes/no binary items so to allow further multivariate analysis by PCA [22].

After the filtering out of ‘very low variance’ items (see Methods) a 120 variables structure was identified. Among the filtered out variables, it is worth noting that from 90 to 100% of women answered YES to items, such as: ‘Do you think is it important the physician show you the care program for the nine months of pregnancy (diagnostic tests, exams…)?’ showing an almost globally shared need of participation to the entire care process. Another item reaching a near 100% YES answer was: ‘Did you have information about alimentation and life style in pregnancy?’ affirming that their first approach with the sanitary personnel was largely satisfactory. This interpretation is further confirmed by the 100% of ‘NO, NEVER’ answers to the question ‘Did you ever have some doubts about the appropriateness of the care received by healthcare professionals?’.

From the above results we can safely affirm the study population is made by women largely satisfied of their experience, so results could be considered in terms of a ‘detailed scale’ analysis of women feeling about their experience not biased by any relevant reason of complaint. The 120 variable set was analysed by OPC variable clustering giving rise to five homogenous groups according to R^2^ metrics (see Methods). S1 Table reports the output of OPC.

The choice of selecting for the further steps of the analysis only the variables showing a relevant level of consistency with other items (singular, poorly classified variables are discarded) increases the robustness of the study [18].

### 2. Opinion (OP) variables correlation structure

The eigenvalue distribution along the principal components of the OP set allowed to choose a six component (OP 1–6) solution as ‘*bona fide’* signal after a scree test (see Table 1).

**Table 1:** Loading pattern for Opinion variables (bolded values point to variables relevant for component meaning, Italics to borderline items).

Variables	OP1	OP2	OP3	OP4	OP5	OP6
AOP	-0.377	0.172	0.215	**-0.402**	0.309	**0.411**
BOP	-0.146	-0.035	-0.347	*-0.366*	**0.667**	0.173
COP	**0.712**	0.099	-0.139	*-0.305*	0.067	0.069
DOP	**-0.606**	-0.041	*0.401*	0.015	-0.054	-0.056
NOP	**0.601**	-0.139	0.271	0.309	0.079	0.234
LOP	0.366	-0.089	0.245	**0.454**	0.387	**0.427**
FOP	-0.225	0.007	**-0.534**	**0.544**	-0.149	0.059
HOP	**0.508**	-0.307	-0.167	-0.179	0.162	**-0.465**
IOP	-0.176	0.034	0.372	**0.402**	**0.499**	-0.295
GOP	-0.147	0.041	**-0.642**	0.198	0.029	0.378
MOP	0.123	-0.273	0.335	-0.179	**-0.455**	**0.452**
POP	0.137	**0.808**	0.148	0.092	0.061	-0.059
QOP	0.204	**0.845**	-0.026	-0.039	-0.158	0.024

**Variable codes**: **AOP** = How much did you need physician during pregnancy?; **BOP** = How much did you need midwife during pregnancy?; **COP** = How much did you think the physician gender is important?; **DOP** = Did you prefer a male or female doctor? (1 = Male, 0 = Female); **NOP** = ‘How should be the perfect doctor/patient relationships? (High values: Mostly based on human relationships; Low Values: Mostly based on technical proficiency; Intermediate Values: Balance between human and technical); **LOP** = ‘How should be the perfect midwife/patient relationships? (High values: Mostly based on human relationships; Low Values: Mostly based on technical proficiency; Intermediate Values: Balance between human and technical); **FOP** = Are you used to verify the doctor or midwife suggestions on Internet?; **HOP** = Do you think midwife alone is sufficient for care?; **IOP** = How much dialogue is important in the patient/doctor(midwife) relation?; **GOP** = How much privacy is important in the relation with healthcare professionals?; **MOP**: How much consideration is important in the relationships with healthcare professionals?; **POP**: How much human virtues of the physician are important?; **QOP**: How much human virtues of the patient are important?.

The six components solution corresponded to a cumulative 67% of total variance explained. In the following we try to assign a meaning to the components taking into consideration their loading profile (the loadings correspond to the Pearson correlation coefficients between variables and components). The bolded values in Table 1 correspond to items relevant for component meaning, the sign of the loadings allows the reader to discriminate between items going in the same or opposite direction along each component, the italics values correspond to borderline variables.

**OP1** explains 15% of total OP set variance and clearly depicts the struggle between ‘nature’ and ‘medicine’ in the birth event. This opposition corresponds to the elevated loadings of the items redounding on the importance of physician gender.

OP1 loading pattern inspection (Table 1) allows to grasp that women assigning a relevant importance to the physician gender (COP) neatly prefer a female doctor (DOP). The preference for a female doctor goes hand-in-hand with the indication of no need of medical doctors (HOP) for birth. Thus high values of OP1 scores point to women most oriented toward the pole ‘Birth is STILL a natural event to be managed in a female-only environment’, while low values of OP1 point toward ‘Birth is NOW a medical act having as main quality factor the professional competence of the healthcare workers’ (see Table 1).

**OP2** (13% of variance explained) has to do with the between patient and physician relation. Women having high values of OP2 give great importance to human values shared by patient and physician (POP and QOP variables), while women with low values of OP2 do not consider this ethical commonality as particularly relevant (see Table 1).

**OP3** (11.4% of variance explained) makes a currently debated opinion trend to emerge: OP3 negatively correlates with the tendency of autonomously checking on the web the validity of the information received from the healthcare professionals (FOP) and with the importance assigned to Privacy (GOP). Low scores of OP3 indicate a low level of faithfulness in healthcare professionals (see Table 1). The OP3 dimension could be crucially important for possible litigations [7–9].

**OP4** (10% explained variance) marks the tendency toward the consideration of birth as a ‘midwife/woman’ affair with no intrusion by the physician. High values of OP4 (see Table 1) show a perceived low importance of the physician role compared to midwife, who in turn is not considered for her technical competences (BOP and AOP) but as a dialoguing partner (IOP) for an empathic personal relation (LOP).

**OP5** (9.5% explained variance) focuses on the need of having a strong relation with midwife during pregnancy and delivery. This relation has to do with both trustiness and commitment. It is worth noting the opposite relation across OP5 between the importance assigned to ‘received consideration’ (MOP) and to ‘need for midwife’ (BOP): women with high values of OP5 (see Table 1) do not care about their own pride (MOP) when in a potentially at risk situation like pregnancy and birth.

**OP6** (8.7% of variance explained) is a dimension correlating self-esteem and pride (MOP) with the importance of a good midwife/patient relation and the recognition of an important role played by the physician (AOP). Positive values of OP6 (see Table 1) point to women who are proud to be part of the technical aspects of the pregnancy and birth, while negative values of OP6 should prefer the lack of any medical intrusion in their lives.

### 3. Evaluation (EVAL) variables correlation structure

PCA applied to the Evaluation field (EVAL) suggests a six components solution as well, globally explaining 51% of total variability (see Table 2). In the following we discuss EVAL component space along the same lines adopted for Opinion components.

**Table 2:** Loading pattern for Evaluation variables (bolded values point to variables relevant for component meaning, Italics to borderline items).

Variables	EVAL1	EVAL2	EVAL3	EVAL4	EVAL5	EVAL6
AVAL	**-0.462**	-0.163	**-0.416**	0.136	*0.309*	*0.326*
BVAL	-0.129	-0.031	-0.122	**0.423**	**0.439**	0.013
CVAL	*0.341*	*0.345*	0.079	-0.065	0.283	-0.179
DVAL	0.375	-0.086	-0.003	-0.109	-0.062	**-0.424**
FVAL	0.131	*0.343*	-0.085	*0.333*	0.145	-0.253
GVAL	0.138	**0.598**	-0.237	-0.187	-0.005	-0.003
HVAL	0.215	**0.446**	0.187	-0.007	0.283	**0.359**
IVAL	0.098	**0.419**	-0.176	0.103	-0.098	**0.567**
JVAL	0.063	0.236	**0.451**	-0.207	-0.281	0.139
KVAL	**0.439**	0.163	**0.529**	0.019	-0.045	0.035
LVAL	**0.525**	-0.033	*0.381*	*0.305*	-0.045	-0.161
MVAL	0.086	-0.031	**0.691**	0.183	0.279	0.261
NVAL	**0.407**	0.024	0.129	**0.407**	-0.187	0.092
OVAL	**0.463**	-0.029	-0.045	**0.402**	*-0.327*	-0.209
PVAL	**0.431**	**0.534**	-0.044	0.064	0.039	0.002
QVAL	*0.395*	**0.451**	-0.148	0.156	**0.411**	0.218
RVAL	0.225	**0.591**	-0.261	-0.242	-0.001	0.202
SVAL	**0.436**	**-0.522**	0.038	0.159	-0.076	0.248
TVAL	-0.023	*0.339*	-0.172	-0.011	**-0.532**	-0.282
UVAL	*0.382*	-0.086	**0.433**	0.085	**0.393**	-0.046
VVAL	**0.613**	-0.259	**-0.395**	-0.071	0.128	0.047
WVAL	**0.598**	-0.011	**-0.412**	0.239	0.077	-0.186
XVAL	**0.762**	-0.116	-0.216	0.096	0.149	-0.022
YVAL	**0.435**	-0.056	-0.108	**-0.549**	0.149	-0.092
ZVAL	0.305	-0.044	0.158	**-0.604**	*0.314*	-0.062
A2VAL	**-0.558**	*0.316*	0.062	0.173	0.221	-0.169
B2VAL	0.291	**-0.452**	-0.123	-0.058	-0.272	0.282
C2VAL	**0.542**	*-0.306*	-0.169	-0.143	-0.105	0.236

**Variables codes**: **AVAL** = ‘Did your physician ever answered to phone calls?’; **BVAL**: ‘During pregnancy did you feel the need of more visits/exams?’; **CVAL** = ‘Did you receive all the necessary information during pregnancy/birth/post-birth periods?’; **DVAL** = ‘Was ever it possible to express your opinion during visits?’; **FVAL** = ‘Which is your evaluation of the technical skills of gynecologist?’; **GVAL** = ‘Which is your opinion of the patience demonstrated by your gynecologist?’; **HVAL**: ‘Which is your opinion about the clarity of the information given by gynecologist?’; **IVAL**: ‘Which is your opinion about the tact demonstrated by gynecologist in the relationships?’; **JVAL** ‘Which is your opinion about the professional ethics of your gynecologist?’; **KVAL**: ‘During your relationships with doctors did you ever felt considered/understood?’; **LVAL**: ‘Did the physician ever made all that it was possible to meet your needs?’; **MVAL** ‘Give a global score to your relationship with gynecologist’; **NVAL** ‘Did the physician made you to participate in the different choices?’; **OVAL**: ‘‘At the end of the birth, post-birth period how much your expectations were met?’; **PVAL, QVAL, RVAL**, are the possible answers to the question ‘Which of the virtues of your gynecologist did you appreciate most?’ correspondent to technical skills, patience, cheerfulness respectively; **SVAL**: ‘Did the midwife ever made all that it was possible to meet your needs?’; **TVAL** ‘Did you feel sometimes inappropriate in your questions and/or judged for your choices? (physician)’; **UVAL** ‘When arrived at the hospital had you to wait a long (*low values*) or short (*high values*) time?’; **VVAL**: ‘Did you think healthcare professionals put effort into establishing a positive human relationship with you?’; **WVAL**: ‘The relational environment you found among healthcare professionals did make you to feel safe?; **XVAL** ‘At admittance time did you feel yourself embraced?’; **YVAL**: ‘During your relationships with midwife did you ever felt considered/understood as well as supported in your decisions?’; **ZVAL:** ‘‘Did you feel sometimes inappropriate in your questions and/or judged for your choices? (midwife)’; **A2VAL** ‘Did you feel the need of more assistance during the first hours after delivery?’; **B2VAL**: ‘Did you judge as sufficient the information and the received assistance after you went back home?’; **C2VAL** ‘Did you receive a psychological support from health care professionals?.

**EVAL1** accounts for 16% of total variance and relates to First Welcome / Warmth. General evaluation variables (KVAL, LVAL, OVAL) are significantly loaded on EVAL1 so this component can be intended as a Global Evaluation of Quality of Care. It is worth noting the variable most loaded on EVAL1 is XVAL (loading = 0.76) that corresponds to the item “At admittance did you feel embraced?”. This implies EVAL1 is a comprehensive evaluation score of the entire process of care with a specific focus on the ‘first impression’. If the woman feels herself embraced at hospital admittance she experiences a general relief that influences her global perspective on the entire healthcare process evaluation. It is worth noting the variable A2VAL (“Did you feel the need of more assistance during the first hours after delivery?”) negatively scales with EVAL1 suggesting a warmer welcome has an enduring effect after the delivery ending up in a lower request of assistance. We prefer the term ‘warmer’ over ‘more efficient’ welcome relying on the fact VVAL (“Did you think healthcare professionals put effort into establishing a positive human relationship with you?”) scales very significantly with EVAL1 at odds with “technical skills” items that have no relevant loadings on the component. EVAL1 expresses the most prominent dimension of evaluation field proposing, on a different perspective, the nature/medicine opposition recognized in OP1.

**EVAL2** accounts for 10% of total variance and focuses on gynecologist’s virtues, both human and technical, with a clear prevalence of human and relational aspects. It is worth noting that positive loadings for gynecologist evaluation go together with negative loading for SVAL: “Did the midwife ever made all that it was possible to meet your needs?” EVAL2 thus accounts for the relative preference for the physician vs. midwife relation.

**EVAL3** accounts for 8% of total variance and has as pivot variable MVAL, the global evaluation of the relation with gynecologist. The separation of human and technical virtues of gynecologist into two different components (EVAL2 and EVAL3 that are each other independent by construction) is a further proof of the main hypotheses set, i.e. the opposition between the two competing nature centered/ technique centered views of the delivery event.

**EVAL4** explains 6.3% of total variance and has two main leading variables: YVAL “During your relationships with midwife did you ever feel considered/understood as well as supported in your decisions?” and ZVAL: ‘‘Did you feel sometimes inappropriate in your questions and/or judged for your choices? (midwife)’. EVAL4 is a mainly relational component rooted on the level of empathy reached with the midwife, that in turn prevents potentially embarrassing situations (ZVAL). It is worth noting BVAL: “During pregnancy did you feel the need of more visits/exams?” has an opposite sign with EVAL4 with respect to YVAL and ZVAL: a better relationship with midwife potentially allows for a decreased need of exams and visits with a neat economic advantage for welfare system.

**EVAL5:** This component explains 6% of variance. It highlights (under a slightly different perspective) the same latent motivations already discussed for EVAL4: the desire of more exams and visits (again BVAL) even in presence of a good evaluation of the received healthcare (positive loading with OVAL). The need of more exams and visits is decreased by the empathy reached with the physician (negative loading of EVAL5 with TVAL ‘Did you feel sometimes inappropriate in your questions and/or judged for your choices? (physician)’). A more empathic relation with gynecologist decreases the desire of new visits and examinations thus exerting an additive effect with EVAL4.

**EVAL6**: This relational component accounts for 5% of total variance and highlights a very peculiar aspect of the analyzed data set: the negative relation existing between the possibility to express a personal opinion during the medical exam (DVAL) and the funny and merry character of the doctor (IVAL). Too joyful and merry doctors tend to inhibit the propensity of the patient to ask too much and to express her personal opinion.

### 4. How opinion affects evaluation

The subdivision of the questionnaire into OP (Opinion) and EVAL (Evaluation) items allows to estimate the importance of general opinions about the patient/health professional in shaping the perception of health care (see Table 3). EVAL and OP spaces were poorly correlated: the maternity experience seems to re-shape preconceived ideas eventually pushing the mother to consider reality independently of her abstract preferences. This situation is potentially very fertile for the planner because allows for a greater effect of organizational and behavioral changes.

**Table 3:** Pearson correlations between OP and EVAL components (bolded values correspond to statistically significant correlations at p < 0.05).

Components	OP1	OP2	OP3	OP4	OP5	OP6
EVAL1	0.107	-0.134	0.078	0.132	**0.282**	**-0.214**
EVAL2	-0.123	-0.064	0.053	0.114	-0.025	**0.257**
EVAL3	0.036	-0.109	-0.067	0.127	0.075	0.136
EVAL4	-0.144	0.172	-0.107	**0.298**	-0.005	-0.028
EVAL5	**0.289**	0.024	-0.018	0.072	0.146	**0.258**
EVAL6	-0.112	-0.135	-0.155	0.033	-0.032	-0.089

### 5. External factors acting as response modifiers

The external variables globally had a minor effect on both opinion and evaluation fields. Neither physical (Height, Weight at the beginning/end of pregnancy) nor demographic (Age, Level of Education, Place of birth, Profession, Hospital) highlighted any relevant effect on both OP and EVAL components.

A statistically significant (Analysis of Variance, F = 5.6, p < 0.02) relation between the type of delivery (Caesarean Section / Spontaneous Delivery) and the first component of evaluation field (EVAL1) was observed. EVAL1 had a mean value of -0.34 in case of Caesarean section (N = 30) versus a mean = 0.17 for spontaneous delivery (N = 59).

This difference goes in the expected direction of a decreased importance assigned to the quality of welcome (pivot variable of EVAL1) by women undergoing a more ‘medically intensive’ procedure like caesarean section compared to spontaneous birth.

The quality of the human relationship with the gynecologist (EVAL2) was significantly influenced (Analysis of Variance, F = 4.23, p < 0.02) by the civil status of the mother expressed in the three classes: married, cohabitee, single. The quality of human relationship with gynecologist linearly increases with the degree of stability of the relation of the woman with her partner, independently by maternal age. The general mood of the mother has a significant effect on EVAL2 (Analysis of Variance, F = 5.17, p < 0.02) with a worse relation with gynaecologist (low values of EVAL2) for worse mood. This largely expected effect of general mood does not explain the effect of civil status given the three civil status classes were practically identical as for general mood score. An ‘increased suspicion’ effect for less stable civil statuses having consequences on the relation with physician could be at work.

## Conclusion

Existing studies on patient satisfaction tend to break it down into its fundamental components so to find a reliable model of maximal satisfaction in the classical form of a ‘target function’. These studies need to cope with subjects widely varying in terms of satisfaction levels so to make it possible to establishing significant correlations between. levels of satisfaction and specific features of the care process.

Here we analyse a completely different situation in which all the women are satisfied by the received care. The goal of the study is to identify “hidden” ideas and feelings orienting the women perception of delivery experience corresponding to the latent dimensions organizing their needs and expectations.

The feelings are strictly personal and are finely tuned among different subjects, so we expect they distribute along gradients correspondent to the different ‘general ideas’ impinging on such a crucial experience as delivery. These ‘general ideas’ have different weights for different women and the only way to detect them is in terms of the correlation pattern these ideas induce on a set of items.

This translates into the emphasis given to items correlation structure: the emerging principal components can be thought as the ‘axes’ endowed with the maximal efficiency in explaining among women differentiation (higher variance) more than consensus. These axes are such ‘general ideas’ and can be considered as the main concepts driving patient healthcare evaluation. The observed results gave a proof-of-concept of our methodological approach allowing clearly identifiable motivation and evaluation principles emerging from the survey. It is worth reminding that the generation of principal components is a totally unsupervised and data-driven process by no means directed by any ‘target function’. Moreover the principal components of each data set (in our case OP and EVAL sets) are each other independent by construction so pointing to linearly independent dimensions.

The nature vs. technique opposition we hypothesized as the main latent concept shaping the women perception of their experience, was recognizable in the loading distribution of both evaluation (EVAL) and opinion (OP) components. Nature/Technique opposition took different forms ranging from the need of “transforming hospital into home” (as expressed by the high importance assigned to welcome and warmth of the hospital), to the “acquiring of both self-consciousness and technical skills by the women”, as expressed by OP3, pointing to a group of women personally checking all the medical acts.

The nature vs. technique struggle appears under different forms in all the extracted components highlighting the complexity and depth of this opposition. The results highlight the crucial role played by the midwife as the link between natural and technical milieu. Our results indicate how women look at midwife as their “natural ally” in their resistance against a totalitarian technical perspective on delivery.

The “fault plane” between nature and technique is a very critical zone for litigation. We demonstrated that women are particularly sensitive to the consideration and attention they receive at their admission in the hospital, as well as to the quality of human relations with midwife. The perceived quality of welcome correlates with a decreased of perceived need of additional care and, more in general, with a more faithful attitude toward the health professionals with an expected effect in the decrease of both welfare costs and litigation. Our results suggest the importance of rename this care’s field as "Shared Agency" [23,24] trying to put into the system not only the obstetrician and gynecologist but also the other figures that appear to have a certain impact on the dynamics of care (in the first place admissions staff and family members). Like any Integrated Act [25], collaboration of the parties involved is a prerequisite for a higher quality of Medical Act (safety, effectiveness and efficiency) having a concrete regulatory value.

## Supporting Information

S1 Table Oblique Principal Component Analysis Output (Variable Selection). (DOCX)

S1 File Questionnaire Structure and items (English and Italian versions). (DOCX)

S1 Dataset Original Data set. (XLS)
