# Supplementary material for: The Influence of Doctor-Patient and Midwife-Patient Relationship in Quality Care Perception of Italian Pregnant Women: An Exploratory Study
Source: PLoS One. 2015 Apr 23;10(4):e0124353. doi: 10.1371/journal.pone.0124353 (PMC4408047; doi:10.1371/journal.pone.0124353)
Supplement: S1 Table — (DOCX) [file pone.0124353.s001.docx]

**Supplementary File : Variable Selection**

The File reports the output of Oblique Principal Component (OPC) Analysis. Bolded items (variables) correspond to the items saved for the subsequent analysis (high values of R-square with their own cluster), Italics mark the items summed up into a synthetic index, all the other items are discarded as singular.

**Oblique Principal Component Cluster Analysis**

Observations 88 PROPORTION 1

Variables 103 MAXEIGEN 0

Orthoblique Initialization

Clustering algorithm converged.

Cluster summary for 5 clusters

Cluster Variation Proportion Second

Cluster Members Variation Explained Explained Eigenvalue

ƒƒƒƒƒƒƒƒƒƒƒƒƒƒƒƒƒƒƒƒƒƒƒƒƒƒƒƒƒƒƒƒƒƒƒƒƒƒƒƒƒƒƒƒƒƒƒƒƒƒƒƒƒƒƒƒƒƒƒƒƒƒƒƒƒƒƒƒƒƒƒƒ

1 11 11 5.677146 0.5161 1.5792

2 26 26 5.405351 0.2079 2.0738

3 26 26 4.896503 0.1883 2.2754

4 15 15 3.250304 0.2167 1.5935

**Total variation explained = 23.93422 Proportion = 0.2324**

R-squared with

ƒƒƒƒƒƒƒƒƒƒƒƒƒƒƒƒƒƒ

Own Next 1-R**2 Variable

Cluster Variable Cluster Closest Ratio Label

ƒƒƒƒƒƒƒƒƒƒƒƒƒƒƒƒƒƒƒƒƒƒƒƒƒƒƒƒƒƒƒƒƒƒƒƒƒƒƒƒƒƒƒƒƒƒƒƒƒƒƒƒƒƒƒƒƒƒƒƒƒƒƒƒƒƒ

Cluster 1 V9G1 0.1105 0.0339 0.9207 V9G1

**V23G1a 0.5952** 0.0098 0.4088 V23G1a

**V23G1b 0.5674** 0.0205 0.4416 V23G1b

**V23G1c 0.3542** 0.0214 0.6599 V23G1c

V23G1d 0.1674 0.0703 0.8956 V23G1d

V32G1c 0.0649 0.0267 0.9608 V32G1c

***V39G1a 0.7727*** 0.0282 0.2340 V39G1a

***V39G1b 0.8359*** 0.0441 0.1717 V39G1b

***V39G1c 0.7014*** 0.0599 0.3177 V39G1c

***V39G1d 0.7936*** 0.0505 0.2174 V39G1d

***V39G1e 0.7140*** 0.0416 0.2984 V39G1e

R-squared with

ƒƒƒƒƒƒƒƒƒƒƒƒƒƒƒƒƒƒ

Own Next 1-R**2 Variable

Cluster Variable Cluster Closest Ratio Label

------------------------------------------------------------------

Cluster 2 **V11G1 0.2207** 0.0520 0.8220 V11G1

V16G1 0.0989 0.0302 0.9291 V16G1

V20G1 0.1219 0.0076 0.8849 V20G1

V23G1e 0.1025 0.0665 0.9614 V23G1e

V23G1g 0.0610 0.0220 0.9601 V23G1g

**V27G1 0.2165** 0.0689 0.8415 V27G1

V30G1 0.1220 0.0304 0.9056 V30G1

V32G1a 0.0228 0.0095 0.9866 V32G1a

V32G1e 0.0987 0.0257 0.9251 V32G1e

**V33G1 0.2107** 0.1182 0.8951 V33G1

**V34G1 0.2481** 0.0347 0.7789 V34G1

V35G1 0.0287 0.0082 0.9794 V35G1

V37G1a 0.0699 0.0076 0.9373 V37G1a

**V41G2 0.3100** 0.0766 0.7472 V41G2

**V44G2 0.2326** 0.0447 0.8032 V44G2

**V45G2 0.2373** 0.0380 0.7928 V45G2

**V50G2 0.4359** 0.0951 0.6235 V50G2

V56G2 0.0459 0.0158 0.9694 V56G2

**V57G2 0.4860** 0.0204 0.5247 V57G2

**V59G2 0.4638** 0.1086 0.6015 V59G2

V60G2 0.1724 0.0179 0.8426 V60G2

**V61G2 0.6073** 0.0849 0.4291 V61G2

V73G3 0.1063 0.0155 0.9078 V73G3

**V86G4 0.2628** 0.0148 0.7483 V86G4

V87G4 0.1143 0.0419 0.9243 V87G4

**V88G4 0.3083** 0.0157 0.7028 V88G4

------------------------------------------------------------------

Cluster 3 V4ASD 0.0284 0.0164 0.9877 V4ASD

V12ASD 0.0880 0.0090 0.9203 V12ASD

V1G1A 0.0429 0.0208 0.9775 V1G1A

V1G1E 0.0490 0.0209 0.9712 V1G1E

V4G1B 0.1492 0.0057 0.8557 V4G1B

V7G1 0.0455 0.0072 0.9615 V7G1

**V24G1b 0.3003** 0.0187 0.7130 V24G1b

V24G1d 0.1116 0.0288 0.9147 V24G1d

**V24G1e 0.2842** 0.0468 0.7509 V24G1e

V24G1f 0.2064 0.0424 0.8287 V24G1f

V24G1g 0.1081 0.0306 0.9200 V24G1g

V28G1 0.0763 0.0708 0.9940 V28G1

**V36G1b 0.2225** 0.0976 0.8616 V36G1b

**V36G1c 0.2883** 0.0361 0.7384 V36G1c

V36Gd 0.1601 0.0202 0.8572 V36Gd

**V37G1c 0.2682** 0.0481 0.7688 V37G1c

**V37G1d 0.4193 0.0306 0.5991 V37G1d**

V40G2a 0.1993 0.0494 0.8423 V40G2a

**V40G2b 0.3146 0.0236 0.7020 V40G2b**

**V40G2c 0.2496** 0.0500 0.7899 V40G2c

V40G2d 0.1371 0.0319 0.8914 V40G2d

**V40G2e 0.3500 0.0061 0.6539 V40G2e**

**V40G2f 0.3977 0.0139 0.6107 V40G2f**

**V40G2g 0.2740** 0.0298 0.7483 V40G2g

V42G2 0.0338 0.0224 0.9883 V42G2

V71G3 0.0918 0.0421 0.9481 V71G3

R-squared with

ƒƒƒƒƒƒƒƒƒƒƒƒƒƒƒƒƒƒ

Own Next 1-R**2 Variable

Cluster Variable Cluster Closest Ratio Label

------------------------------------------------------------------

Cluster 4 V1ASD 0.1837 0.0095 0.8241 V1ASD

**V8ASD 0.5491** 0.0123 0.4565 V8ASD

**V10ASD 0.4874**  0.0329 0.5301 V10ASD

V1G1B 0.1115 0.0282 0.9143 V1G1B

**V1G1C 0.3589** 0.0032 0.6432 V1G1C

V1G1D 0.1592 0.0122 0.8511 V1G1D

V3G1 0.0667 0.0127 0.9454 V3G1

VC5G1 0.1893 0.0376 0.8424 VC5G1

V6G1 0.1084 0.0167 0.9068 V6G1

V14G1 0.0392 0.0091 0.9697 V14G1

V17G1 0.1640 0.0305 0.8624 V17G1

**V18G1 0.2267** 0.0153 0.7853 V18G1

**V32G1d 0.2243** 0.0313 0.8008 V32G1d

**V36G1a 0.2131** 0.0357 0.8160 V36G1a

V37G1b 0.1691 0.0908 0.9139 V37G1b

------------------------------------------------------------------

Cluster 5 PREQ 0.0627 0.0152 0.9517 PREQ

**V5ASD 0.2823 0.0447 0.7513 V5ASD**

**V6ASD 0.3041 0.0380 0.7235 V6ASD**

**V4G1A 0.3487 0.0057 0.6550 V4G1A**

V4G1C 0.1038 0.0629 0.9564 V4G1C

V12G1 0.1343 0.1282 0.9931 V12G1

**V13G1 0.2924 0.0018 0.7089 V13G1**

V19G1 0.0668 0.0503 0.9827 V19G1

V23G1f 0.0734 0.0190 0.9446 V23G1f

V24G1a 0.1931 0.0605 0.8589 V24G1a

V24G1c 0.0802 0.0059 0.9253 V24G1c

V26G1 0.1549 0.0475 0.8872 V26G1

V29G1a 0.0932 0.0121 0.9179 V29G1a

V32G1b 0.0799 0.0052 0.9248 V32G1b

V49G2 0.0832 0.0387 0.9538 V49G2

V62G2 0.1381 0.0403 0.8981 V62G2

V63G2 0.0923 0.0307 0.9364 V63G2

**V66G3 0.2801 0.0740 0.7775 V66G3**

**V67G3 0.4374 0.0420 0.5873 V67G3**

V72G3 0.1202 0.0158 0.8939 V72G3

**V73G3BIS 0.3635 0.2099 0.8056 V73G3BIS**

**V74G3 0.3139 0.0686 0.7367 V74G3**

V75G3 0.1449 0.0087 0.8626 V75G3

**V79G3 0.2459** 0.0642 0.8058 V79G3

**V83G4 0.2158** 0.0313 0.8095 V83G4
