# Supplementary material for: The Influence of Doctor-Patient and Midwife-Patient Relationship in Quality Care Perception of Italian Pregnant Women: An Exploratory Study
Source: PLoS One. 2015 Apr 23;10(4):e0124353. doi: 10.1371/journal.pone.0124353 (PMC4408047; doi:10.1371/journal.pone.0124353)
Supplement: S1 File — (DOCX) [file pone.0124353.s002.docx]

**Supplementary File**

**Questionnaire structure:**

*General information*: As described in the text, the questionnaire is separated into opinion (suffix **OP**) and evaluation (suffix **VAL**) questions. Added to the questionnaire (see main text and actual data set) are external demographic variables ; Hospital, Delivery, Age , Place of Birth, Place of Residence, Height , Weight, Marital status, school degree, school degree of the partner and a question about mood (**MOOD** variable, going from 0 to 5, where higher values correspond to a better mood). The respondents did not know if a specific item is **OP** or **VAL** so not to be biased by any *a priori* interpretation. Variables are expressed in terms of rank scores, there are four levels of ranking (**1**) Scores going from 0 to 5; (**2**) Scores going from 0 to 2; (**3**) Binary Yes = 1; No = 0; (**4**) Variables summing up two (**NOP, LOP**,**POP)** or four (**QOP**) items and thus ranging from 0 to 10 and 0 0 to20 respectively. Given PCA was computed on the basis of the eigenvectors of the correlation matrix the different variability range had no effect on the items weight on the solution.

All the items had most ‘affirmative’ scores (i.e. phrases like ‘Very Much’, ‘Mostly’; ‘Every time’..) corresponding to higher ranks and consequently most ‘negative’ answers correspond to lowest values. In the cases where the ‘positive’ / ‘negative’ direction cannot be taken as such but necessitates further explanation the ‘cline’ at the basis of the scores are made explicit.

Each variable has (in parentheses) the formalization class they pertain.

**English version**

**Opinion Variables:**

**AOP (3)** = Did you need physician during pregnancy ?;

**BOP** **(3)**= Did you need midwife during pregnancy ?;

**COP (1)**= How much did you think the physician gender is important ?;

**DOP (3)**= Did you prefer a male or female doctor ? (1=Male, 0=Female);

**NOP (4)**= ‘How should be the perfect doctor/patient relationships ? (High values: Mostly based on human relationships; Low Values : Mostly based on technical proficiency; Intermediate Values: Balance between human and technical);

**LOP (4)** = ‘How should be the perfect midwife/patient relationships ? (High values: Mostly based on human relationships; Low Values : Mostly based on technical proficiency; Intermediate Values: Balance between human and technical);

**FOP (3)** = Are you used to verify the doctor or midwife suggestions on Internet ?;

**HOP** **(3)** = Do you think midwife alone is sufficient for care ? **;**

**IOP** **(1)** = How much dialogue is important in the patient/doctor(midwife) relation ?;

**GOP (1)** = How much privacy is important in the relation with healthcare professionals ?;

**MOP** **(1)** = How much consideration is important in the relationships with healthcare professionals? **POP** **(4) =** How much human virtues of the physician are important ? ;

**QOP**  **(4)** = How much human virtues of the patient are important ?.

**Evaluation Variables**:

**AVAL (3)** = ‘Did your physician ever answered to phone calls ?’

**BVAL (3)** : ‘During pregnancy did you feel the need of more visits/exams ?

**CVAL** **(3)** = ‘Did you receive all the necessary information in pregnancy/birth/post-birth periods ?’; **DVAL** **(2)** = ‘Was ever it possible to express your opinion during visits ?’;

**FVAL** **(2)** = ‘Which is your evaluation of the technical skills of gynecologist ?’;

**GVAL (2)** = ‘Which is your opinion of the patience demonstrated by your gynecologist ?’;

**HVAL** **(2)** ‘Which is your opinion about the clarity of the information given by gynecologist ?’

**IVAL** **(3)** ‘Which is your opinion about the tact demonstrated by the gynecologist ?’;

**JVAL** **(3)**‘Which is your opinion about the professional ethics of your gynecologist ?

**KVAL (1)** ‘During your relationships with doctors did you ever felt considered/understood ?’;

**LVAL (1)** : ‘Did the physician ever made all that it was possible to meet your needs ?’

**MVAL** **(1)**‘Give a global score to your relationship with gynecologist’;

**NVAL (1)** ‘Did the physician made you to participate in the different choices ?’

**OVAL (1)**’At the end of the birth, post-birth period how much your expectations were met ?’;

**PVAL, QVAL, RVAL**, **(3)** are the possible answers to the question ‘Which of the virtues of your gynecologist did you appreciate most ?’ correspondent to technical skills, patience, cheerfulness respectively (1 corresponds to a mark on the corresponding virtue, the respondent can give multiple answers, i.e. to score 1 at different items)

**SVAL** **(1)**: ‘Did the midwife ever made all that it was possible to meet your needs ?’;

**TVAL (1)** ‘Did you feel sometimes inappropriate in your questions and/or judged for your choices? (physician)

**UVAL (1)** ‘When arrived at the hospital had you to wait a long (*low values*) or short (*high values*) time ?’;

**VVAL(1)** ‘Did you think healthcare professionals put effort into establishing a positive human relationship with you ?’

**WVAL (1)** : ‘The relational environment you found among healthcare professionals did make you to feel safe ?

**XVAL (1)** ‘At admittance time did you feel yourself embraced ?’

**YVAL (1)**: ‘Did you ever felt considered/understood as well as supported in your decisions ?’(midwife)

**ZVAL (1)** ‘‘Did you feel sometimes inappropriate in your questions and/or judged for your choices? **A2VAL (3)** ‘Did you feel the need of more assistance during the first hours after delivery ?’

**B2VAL (3)** : ‘Did you judge as sufficient the information and the received assistance after you went back home ?’;

**C2VAL (1)** ‘Did you receive a psychological support from health care professionals ?

**Italian Version**

**Variabili di Opinione:**

**AOP (3) =** Ha sentito il bisogno di consultare il medico durante la gravidanza ?

**BOP (3)** = Ha sentito il bisogno di consultare l’ostetrica durante la gravidanza ?

**COP (1)** = Quanto ritiene importante il genere del medico ?

**DOP (3)** = Preferirebbe un medico di genere maschile o femminile ?

**NOP (4)** = Quale dovrebbe essere la relazione ideale tra medico e paziente ? (Valori elevati: Basata prevalentemente sul rapporto umano; Valori Bassi: Basata prevalentemente sulla professionalità; Valori Intermedi: Entrambi gli aspetti sono ugualmente rilevanti).

**LOP (4)** = Quale dovrebbe essere la relazione ideale tra ostetrica e paziente ? (Valori elevati: Basata prevalentemente sul rapporto umano; Valori Bassi: Basata prevalentemente sulla professionalità; Valori Intermedi: Entrambi gli aspetti sono ugualmente rilevanti) .

**FOP (3)** = E’ solita verificare i consigli di medico e/o ostetrica su Internet ?

**HOP (3)** = Ritiene che basti la sola ostetrica per la cura delle partorienti ?

**IOP (1)** = Quanto ritiene importante il dialogo nella relazione tra medico (ostetrica) e paziente ?

**GOP (1)** = Quanto reputa importante il concetto di ‘privacy’ nei rapporti con i sanitari ?

**MOP (1)** = Quanto reputa importante la considerazione nei rapporti con i sanitari ?

**POP (4)** = Quanto reputa importanti le virtù umane del medico ?

**QOP (4)** = Quanto reputa importanti le virtù umane della paziente ?

**Variabili valutative**

**AVAL (3)** = Il suo medico ha sempre risposto alle sue telefonate ?

**BVAL (3)** = Durante la gravidanza ha sentito il bisogno di un maggior numero di visite/esami ?

**CVAL (3)** = Ritiene di aver ricevuto tutta l’informazione di cui aveva bisogno durante la gravidanza/il parto/il post parto ?

**DVAL (3)** = E’ sempre stato possibile esprimere la sua opinione durante le visite ?

**FVAL (3)** = Quanto ritiene importanti le abilità tecniche del ginecologo ?

**GVAL (2)** = Quale è la sua opinione sulla misura di pazienza dimostrata dal suo ginecologo?

**HVAL (2)** = Quale è la sua opinione riguardo alla chiarezza dell’informazione fornita dal ginecologo?

**IVAL (3)** = Quale è la sua opinione riguardo al tatto dimostrato dal suo ginecologo?

**JVAL (3)** = Quale è la sua opinione riguardo all’etica professionale dimostrata dal suo ginecologo?

**KVAL (1)** = Durante i suoi rapporti con i medici si è sempre sentita considerata/compresa?

**LVAL (1)** = Il medico ha sempre fatto il possibile per venire incontro ai suoi bisogni?

**MVAL (1)** = Fornisca una valutazione complessiva della sua relazione con il ginecologo.

**NVAL (1)** = Il medico la ha resa partecipe delle diverse scelte possibili?

**OVAL (1)** = Al termine della sua esperienza (parto e post-parto) in che misura le sue attese sono state rispettate?

**PVAL, QVAL, RVAL** **(3)** = Risposte multiple alla domanda ‘Quale delle virtù del suo ginecologo ha maggiormente apprezzato ?’ : abilità tecnica, pazienta, gentilezza. La risposta può coinvolgere più di una virtù, in tal caso si risponda sì a più di una domanda.

**SVAL (1)** = L’ostetrica ha sempre fatto tutto il possibile per venire incontro ai suoi bisogni?

**TVAL (1)** = Qualche volta si è sentita inappropriata a causa di qualche domanda e/o giudicata per le sue scelte? (medico)

**UVAL (1)** = Dopo essere arrivata in ospedale ha dovuto attendere a lungo (punteggi bassi) o è stata accolta velocemente (punteggi alti)?

**VVAL(1)** = Ritiene che i sanitari si siano impegnati a stabilire una positiva relazione umana?

**WVAL (1)** = Le relazioni che ha osservato tra i sanitari la hanno fatta sentire tranquilla?

**XVAL (1)** = Entrando in ospedale si è sentita accolta?

**YVAL (1)** = Qualche volta si è sentita inappropriata a causa di qualche domanda e/o giudicata per le sue scelte? (ostetrica)

**ZVAL (1)** = Si è sentita generalmente inappropriata per le sue richieste e/o giudicata ?

**A2VAL (3)** = Ha sentito il bisogno di una maggiore assistenza durante le prime ore dopo il parto?

**B2VAL (3)** = Ha giudicato sufficiente l’informazione e l’assistenza ricevuta una volta tornata a casa?

**C2VAL (1)** = Ha ricevuto supporto psicologico da parte dei sanitari ?
